# A SWELL time to develop the molecular pharmacology of the volume-regulated anion channel (VRAC)

**DOI:** 10.1080/19336950.2022.2033511

**Published:** 2022-02-03

**Authors:** Eric E. Figueroa, Jerod S. Denton

**Affiliations:** a Department of Physiology, University of California San Francisco, San Francisco, CA, USA; b Department of Pharmacology, Vanderbilt University, Vanderbilt Institute of Chemical Biology, Nashville, TN, USA; c Department of Anesthesiology, Vanderbilt University Medical Center, Nashville, TN, USA

**Keywords:** VRAC, LRRC8, Pranlukast, zinc pyrithione, Zafirlukast

## Abstract

Newly emerging roles of LRRC8 volume-regulated anion channels (VRAC) raise important questions about the therapeutic potential of VRAC in the treatment of epilepsy, type 2 diabetes, and other human diseases. A critical barrier to evaluating whether VRAC represents a viable drug target is the lack of potent and specific small-molecule inhibitors and activators of the channel. Here we review recent progress in developing the molecular pharmacology of VRAC made by screening a library of FDA-approved drugs for novel channel modulators. We discuss the discovery and characterization of cysteinyl leukotriene receptor antagonists Pranlukast and Zafirlukast as novel VRAC inhibitors, and zinc pyrithione (ZPT), which apparently activates VRAC through a reactive oxygen species (ROS)-dependent mechanism. These ongoing efforts set the stage for developing a pharmacological toolkit for probing the integrative physiology, molecular pharmacology, and therapeutic potential of VRAC.

## Overview of the volume-regulated anion channel (VRAC)

A cell’s ability to regulate its volume in response to osmotic stress arose early in evolution and allowed complex organisms to escape the primordial ocean and invade the land [1–5]. In vertebrate cells, osmotic cell swelling activates a complex process known as regulatory volume decrease, or RVD, where ion channels and transporters mediate K^+^, Cl^−^, organic osmolyte, and water efflux that returns cell volume back to normal [6]. A key player in RVD is the volume-regulated anion channel, or VRAC [7]. VRAC is also known as the volume-sensitive organic osmolyte-anion channel (VSOAC) owing to its role in transporting osmolytes such as taurine glutamate. However, we will use the more common name, VRAC, throughout this review.

After more than 30 years of studying VRAC biophysical, regulatory, and pharmacological properties in diverse cell types, the genes encoding the channel were discovered by two independent laboratories using similar genome-wide, siRNA knockdown, high-throughput screening platforms (discussed below) [8,9]. The cell-based screening assay utilizes a halide-sensitive yellow-fluorescent protein (YFP) variant that undergoes fluorescence quenching as iodide moves through VRAC and into the cytoplasm where YFP is expressed. Knock-down of the leucine-rich repeat containing 8A (*LRRC8A*) gene reduced YFP quenching by iodide, indicating that LRRC8A, also known as SWELL1 [8,9], is an essential subunit of the VRAC complex. Other members of the gene family are *LRRC8B, LRRC8C, LRRC8D*, and *LRRC8E*, none of which give rise to channel activity when expressed alone; however, when co-expressed with LRRC8A, *LRRC8C, LRRC8D*, or *LRRC8E* gives rise to swelling-activated anion channels with distinctive functional properties [8,10]. Cryo-electron microscopy evidence indicates that LRRC8A and LRRC8D form homohexamers when expressed alone in heterologous expression systems lacking other LRRC8 subunits [9,11–15].

The ability to silence the function of all VRAC isoforms by deleting *LRRC8A* provides a convenient way to generate knockout mice and cell lines lacking VRAC activity. This approach has been used widely to generate new insights into the role of VRAC in immune cell function, astrocyte physiology, sperm development, cell signaling in adipocytes, glucose sensing and insulin release by pancreatic beta cells, vascular smooth muscle cell signaling, and cancer drug transport [16–18]. The reader is referred to several recent review articles covering these topics in greater detail [7,19–21]. In some cases, these new discoveries have implicated VRAC as novel therapeutic targets for disease. For example, knockout of LRRC8A specifically in astrocytes protects from brain injury following ischemic stroke, likely due to the channel’s role in glutamate release and excitotoxic neuronal cell death. This suggests VRAC might represent a novel drug target for stroke and other diseases associated with neuronal excitotoxicity. In pancreatic beta cells, VRAC enhances insulin secretion following glucose uptake suggesting the channel could be a novel drug target for type 2 diabetes. In support of this idea, Sah and colleagues recently reported that a small molecule that enhances the stability of VRAC in the cell membrane promotes glucose responsiveness and insulin release from beta cells [22]. Whether or not a frank activator of VRAC would have similar effects is not yet known.

Critically evaluating the therapeutic potential and safety of targeting VRAC for treating stroke, diabetes, and other diseases will require potent and selective channel modulators with suitable metabolic and pharmacokinetic profiles to enable channel modulation *in vivo*. However, the current molecular pharmacology of VRAC falls far short of this requirement, consisting mostly of weak and nonspecific chloride channel blockers that have myriad off-target activities. Below, we provide a brief overview of the current pharmacology of VRAC and then discuss ongoing efforts to develop a new generation of channel modulators.

## Most VRAC inhibitors are neither potent nor specific

Many of the inhibitors that have been used to study VRAC function were discovered serendipitously while searching for blockers of other anion transport pathways [23–27]. Classical anion transporter/channel inhibitors such as SITS (4-acetamido-4’-isothio-cyanato-2,2’-stilbenedisulfonic acid), DIDS (4,4’-diisothiocyano-2,2’-stilbenedisulfonic acid), NFA (niflumic acid), FFA (fluflemic acid), NPPB (5-nitro-2-(3-phenylpropylamino)benzoic acid), DPC (diphenylamine-2-carboxylate), 9-AC (9-Anthracenecarboxylic acid), NPA (N-phenylanthracillic acid), inhibit VRAC with IC_50_s in the single to 100s of micromolar range [28–34], but have off-target effects on numerous other transport pathways.

**Figure 1.**
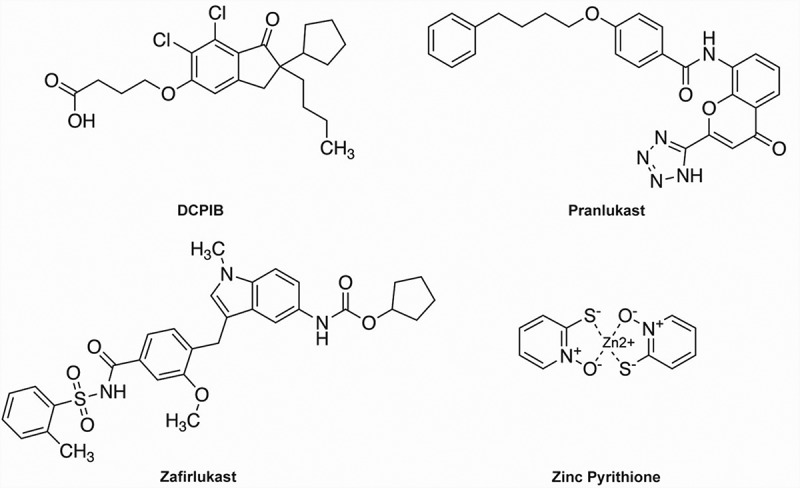
Chemical structures of DCPIB, Pranlukast, Zafirlukast, and ZPT.

The most potent and specific inhibitor of VRAC known to date is the ethacrynic-acid derivative DCPIB (4-(2-butyl-6,7-dichlor-2-cyclopentyl-indan-1-on-5-yl)oxybutyric acid) [35] (Figure 1). DCPIB has been shown to inhibit VRAC in numerous cell types, including HEK293 cells, HELA cells, HCT116 cells, calf bovine pulmonary artery endothelial cells, mouse astrocytes, *Xenopus* oocytes, Guinea-pig atrial cardiomyocytes, and rat pancreatic beta cells, in a voltage-independent, fully reversible manner with an IC_50_ in the range of 2–5 μM [31,35–41]. DCPIB is preferred over other VRAC inhibitors due to its specificity toward VRAC over other Cl^−^ channels such as CFTR, CaCCs, CLCs, Maxi-Cl, and PAC [33,35,42,43]. However, it has been shown to have several off-target effects inward rectifier K^+^ channels, tandem-pore K^+^ channels, H^+^/K^+^-ATPase, connexin hemichannels, glutamate transporter GLT-1, and inhibits mitochondrial respiration [44–48].

The K^+^-Cl^−^ cotransporter antagonist, R-(+)-[(2-n-butyl-6,7-dichloro-2-cyclopentyl-2,3-dihydro-1-oxo-1 H-inden-5-yl)-oxy] acetic acid (DIOA) is structurally related to DCPIB, only differing in the length of its oxy-carbonic acid group (DCPIB has an extra carbon in the linker). DIOA inhibits VRAC in rat thymocytes [49] and HeLa cells with an IC_50_ of 20 mM [33]. The differences in potency between DCPIB and DIOA indicate the length of the oxy-carbonic acid group is likely important for VRAC inhibition.

There are several other compounds that are known to inhibit VRAC but have been used less frequently. For example, the acidic di-aryl urea compound NS3728 inhibits VRAC with an IC_50_ of 400 nM but has off-target effects on calcium-activated Cl^−^ channels [30,50]. The natural phenol, phloretin inhibits VRAC in a voltage-independent and reversible manner with an IC_50_ of 30 μM [51]. Inhibition is only observed when phloretin was applied extracellularly; intracellular application through the pipette solution did not inhibit VRAC [51]. Phloretin also inhibits CFTR, various cation channels, aquaporins, and transporters [51]. The estrogen receptor modulator, tamoxifen, has been shown to inhibit VRAC in astrocytes, endothelial cells, epithelial cells, macrophages, fibroblasts, mouse cortical collecting duct cells with IC_50_ in the low single-micromolar range [29,30,52–57], but interestingly does not inhibit VRAC in neurons [58–60]. Finally, the gap junction/hemichannel inhibitor, carbenoxolone (CBX), inhibits VRAC with an IC_50_ of 15 μM [61], potentially through interactions with the extracellular side of the channel [62]. Unfortunately, none of these VRAC inhibitors are potent or selective enough for evaluating the druggability and therapeutic potential of VRAC for treating diseases.

## A high-throughput screening assay for discovering novel VRAC modulators

A major technological hurdle to discovering new chemical probes of VRAC is the low throughput of manual patch clamp electrophysiology, which is too slow and labor intensive to enable large compound library screening. And although high-throughput electrophysiology instruments exist and are widely used in the pharmaceutical industry, this technology is generally prohibitively expensive for library screening in academic laboratories. The Verkman laboratory developed a series of genetically encoded, yellow-fluorescent protein (YFP)-based anion sensors that effectively overcame these barriers. They developed a double mutant (YFP-H148Q/I152L) that exhibits bright, stable fluorescence that is rapidly quenched when iodide is transported into the cell by a chloride channel or transporter of interest [63,64]. David Weaver and colleagues further optimized temperate stability and fluorescence properties of YFP by introducing a third mutation (YFP-F46L/H148Q/I152L) [65]. YFP-quenching assays have been used extensively the develop the pharmacology of the GABA_A_ receptor, CFTR, calcium-activated chloride channels [66], and was key in discovering the genes that encode VRAC [8,9].

**Figure 2.**
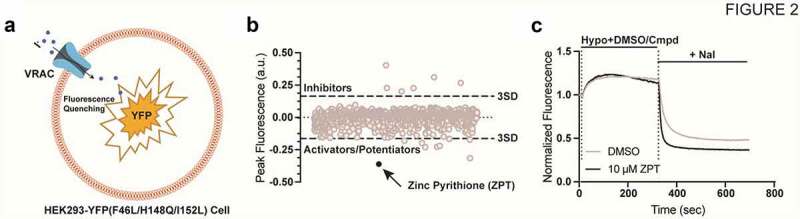
(a) cartoon representation of a HEK293-YFP(F46L/H148Q/I152L) cell used in the YFP quenching assay. HEK293 cells are engineered to constitutively express YFP(F46L/H148Q/I152L). Endogenous VRAC is activated by a hypotonic challenge allowing iodide influx and subsequent YFP quenching. (b) scatter plot of the 1,184 FDA drug compounds screened. A 3 standard deviation from the mean cutoff was implicated to identify compounds as “hits.” Compounds that slowed the rate of fluorescence quenching had positive values, and compounds that increased fluorescence quenching had negative values. (c) representative traces of YFP-quenching assay with 10 µM ZPT. Traces were normalized to mean of five baseline fluorescent measurements taken at the start of the experiment. Hypotonic solution (130 mOsm) containing DMSO (vehicle) or drug compound was added 10s after the start of the experiment. After 5-min incubation period, 100 mM NaI is added and fluorescence quenching is observed. Fluorescence measurements were taken throughout the entirety of the experiment. *Figure is adapted from Figueroa and Denton 2021. AJP-Cell Physiology.*

Figure 2(a) illustrates how the YFP-quenching assay can used to report endogenously expressed VRAC activity in a small-molecule library screen. Cells stably expressing YFP-F46L/H148Q/I152L are plated in black-walled, clear-bottomed, 384-well assay plates and cultured overnight to establish an adherent cell monolayer. The following day, the cell culture media is removed from the plate and exchanged for isotonic buffered saline. The cells are subsequently treated with hypotonic buffer containing compounds or solvent control (e.g. DMSO) to induce cell swelling and VRAC activation, and then treated with iodide-containing buffer to induce VRAC-dependent iodide influx and YFP quenching. Small molecules that inhibit or activate VRAC should reduce or enhance the rate and extent of iodide-induced quenching, respectively.

## Discovery of Pranlukast and Zafirlukast as novel VRAC inhibitors

We employed the YFP-F46L/H148Q/I152L-quenching assay to screen a library of 1,184 FDA-approved drugs for novel modulators of native VRAC expressed endogenously in HEK-293 cells. We selected this library for a pilot screen for several reasons, including its relatively small size, the potential of identifying compounds with drug metabolism and pharmacokinetic properties that are suitable for *in vivo* experiments, and possibility of identifying drugs that could be repurposed for exploring the therapeutic potential of VRAC in preclinical models.

The most potent inhibitor identified in the FDA library screen was Pranlukast (Figure 1), a cysteinyl leukotriene 1 (CysLT1) receptor antagonist used to inhibit bronchospasms in the treatment of asthma [67,68]. Pranlukast is highly selective for CysLT1 over CysLT2 receptors with IC_50_ values of 23 nM and 3,620 nM, respectively [69,70]. In whole-cell patch clamp experiments on HEK-293 cells, Pranlukast inhibited swelling-activated VRAC currents dose-dependently with an IC_50_ of ~3 µM and a maximal inhibitory efficacy of 50% at 10 µM. Inhibition was voltage-independent, occurred with a time constant of ~60 sec, was fully reversible, and was associated with changes in current inactivation kinetics at +120 mV. Interestingly, the structurally distinct CysLT1 receptor antagonist, Zafirlukast (Figure 1), also inhibited VRAC, albeit with an IC_50_ of ~17 µM and nearly 100% efficacy at 100 µM.

The discovery that two structurally distinct CysLT1 receptor antagonists inhibit VRAC activity raised the possibility that VRAC inhibition occurred indirectly through modulation of leukotriene signaling in HEK-293 cells. In support of this idea, Holm and colleagues had previously shown that 60 µM Zafirlukast inhibits RVD and release of the organic osmolyte, taurine, following hypotonic cell swelling of A549 lung epithelial cells [71]. Taken together with their additional observation that an inhibitor of 5-lypoxygenase, the major source of inflammatory leukotrienes that cause asthma, also inhibits RVD and taurine release, led the authors to propose a model in which osmotic cell swelling stimulates the release of leukotrienes which, in turn, signal through CysLT1 to promote VRAC activation and RVD. According to their model, antagonists of CysLT1 should inhibit swelling-induced VRAC activation, as observed in our study [72]. We therefore set out to determine if Pranlukast-dependent inhibition of VRAC in HEK-293 cells is dependent on leukotriene signaling.

CysLT1 is a Gq-coupled GPCR that leads to intracellular calcium release following receptor stimulation, phospholipase C activation, and liberation of IP_3_ from membrane PIP_2_. Thus, if Pranlukast inhibits VRAC indirectly through antagonism of leukotriene signaling, the molecular components of this pathway should be functionally expressed in HEK-293 cells. However, we found that *CYSLTR1* mRNA is not detectably expressed in HEK-293 cells, confirming expression data published on The Human Protein Atlas database (www.proteinatlas.org). Additionally, although acetylcholine was able to induce intracellular calcium release through stimulation of Gq-coupled M3 muscarinic receptors endogenously expressed in HEK-293 cells, the high-affinity CysLT1 agonist, LTD4, neither induced calcium release nor promoted VRAC activation under isotonic conditions. Taken together, these data indicate that the CysLT1 signaling pathway is not functionally expressed in HEK-293 cells and that the inhibitory effects of Pranlukast and Zafirlukast on VRAC occur independently of leukotriene signaling.

## Discovery of zinc pyrithione (ZPT) as a positive modulator of VRAC activity

The anti-dandruff, anti-fouling agent zinc pyrithione (ZPT) (Figure 1) was found in the FDA library screen to dose-dependently increase the rate and extent of YFP (F46L/H148Q/I152L) quenching (Figure 2(b,c)) with an EC50 of 5.7 µM, suggesting the compound acts as a positive modulator of VRAC currents expressed in HEK-293 cells. This was confirmed in whole-cell patch clamp experiments, which demonstrated that ZPT not only potentiates the rate of cell swelling-induced VRAC currents in HEK-293 cells and HCT116 cells but also activates VRAC currents that have reached steady state after swelling and in the absence of swelling.

Given that ZPT is the first-known small-molecule activator of VRAC, understanding its mechanism of action could provide important insights into channel structure–function relationships and regulatory mechanisms. When dissolved in an aqueous buffer, ZPT can dissociate into Zn^2+^ and pyrithione or remain as a ZPT complex, prompting us to examine the effects of each of these components on VRAC activity. Bath application of free Zn^2+^ or free pyrithione failed to activate VRAC in patch clamp experiments, whereas pre-mixing the two components before cell treatment led to channel activation in the absence of hypotonic cell swelling. This observation supports a model in which the Zn^2+^-pyrithione complex mediates the channel activatory response to ZPT.

The ZPT complex could activate VRAC through at least two distinct molecular mechanisms, namely, 1) direct ligand interactions with the channel protein or 2) modulation of cell signaling pathways that regulate VRAC. Although there is currently no experimental evidence to support a direct mechanism of action, the ZPT complex is known to potentiate the activity KCNQ7 (Kv7.2) potassium channels, potentially through ligand interactions with S5 and S6 transmembrane domains. To address an indirect mechanism, we explored wither the effects of ZPT on VRAC are mediated through a reactive oxygen species (ROS)-dependent processes since ZPT induces ROS production in some cell types [73,74] and ROS is known to activate VRAC currents [75–77]. Pre-treatment of cells with the ROS scavenger N-acetylcysteine (NAC) or NAD(P)H inhibitor diphenylene-iodonium (DPI) dramatically reduced the effects of ZPT on VRAC, suggesting the ZPT complex activates the channel indirectly via ROS. There is an emerging literature that further supports such a mechanism. For example, LRRC8A co-localizes and co-immunoprecipitates with the NAD(P)H subunits Nox2, Nox4, and p22phox. ROS induced with chloramine-T activates LRRC8A/C channels through oxidation of intracellular cysteine residues but inhibits LRRC8A/D channels through complex mechanisms that are not yet resolved [78]. It will be important to determine if the effects of ZPT are LRRC8-subunit and cell-type dependent and identify amino acid residues that mediate ZPT’s effects on VRAC activity. Furthermore, considering that ZPT is known to induce apoptotic cell death in some cell types [73,74,79,80], and that VRAC plays a key role in apoptotic cell shrinkage [81], future studies should determine if VRAC is part of ZPT’s therapeutic mechanism of action.

## Use of novel LRRC8 chimeras for probing small-molecule mechanism of action

Whether Pranlukast, Zafirlukast, and ZPT modulate VRAC activity through direct interactions with the channel protein or indirectly via modulation of regulatory-signaling pathways is currently unknown. A commonly used approach to study the molecular pharmacology of an ion channel involves characterizing how engineered mutations alter the pharmacology of the channel in a heterologous expression system. However, VRAC presents at least two unique challenges for studying structure–pharmacology relationships using this approach. First, the *LRRC8* gene family is ubiquitously expresses in vertebrate cells, including commonly used expression systems such as immortalized cells lines and *Xenopus laevis* oocytes, thus, the “background” expression of endogenous wild-type subunits could potentially mask the effects of engineered mutations on heterologously expressed subunits. This limitation can be circumvented with the judicious use of cell lines in which individual or all 5 *LRRC8* genes have been knocked out using CRISPR-mediated technology [8]. A second challenge is that the stoichiometry and molecular arrangement of subunits in a native channel is unknown and possibly variable among cell types [7]. It is conceivable and even likely that the effect of an engineered mutation on channel pharmacology will be influenced by the number and position of mutated subunits present in a channel complex. One solution is to study the pharmacology in homomeric channels comprised one type of subunit. Kern et al. used single-particle cryo-electron microscopy to solve the co-structure of LRRC8A and the best-in-class VRAC inhibitor DCPIB [12]. They observed that DCPIB binds to arginine 103 (R103) of LRRC8A to block the narrowest part of the extracellular pore mouth via a “cork in a bottle” mechanism.

**Figure 3.**
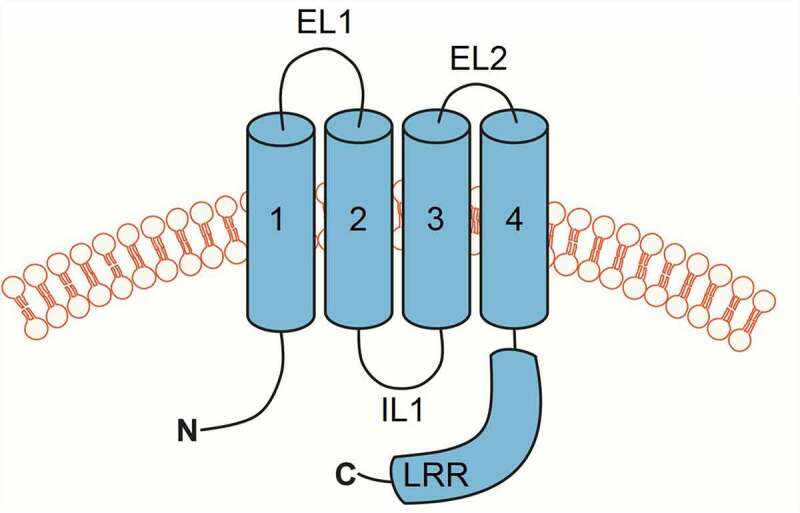
**Cartoon depiction of LRRC8 subunit membrane topology**. Transmembrane domains are numbered 1–4. Extracellular loop domains 1 (EL1) and 2 (EL2), intracellular loop (IL), and leucine-rich repeat (LRR) domains are also shown.

Despite several cryo-EM structures of LRRC8A homohexameric channels being published [11–15], the functional, regulatory, and pharmacological properties of LRRC8A homomeric channels were only recently reported [41] due to the absence of channel activity under normal recording conditions. Unlike native VRAC currents, LRRC8A channels are poorly activated by low intracellular ionic strength and are insensitive to osmotic cell swelling under normal intracellular ionic strength conditions. However, combining low intracellular ionic strength and cell swelling gives rise to modest channel activity, enabling characterization of LRRC8A pore properties. One of the most surprising characteristics of LRRC8A homomeric channels is its weak and voltage-dependent block by DCPIB, which is in striking contrast to the strong and voltage-independent block of native VRAC currents. As predicted from the LRRC8A-DCPIB co-structure [12], mutation of R103 to phenylalanine (R103F) abolished DCPIB-dependent inhibition of LRRC8A but had no effect on DCPIB block of LRRC8A (R103F)/LRRC8C heteromeric channels. Furthermore, a chimeric channel comprising mostly LRRC8C and a short 25-amino acid stretch of intracellular loop 1 (Figure 3) from LRRC8A (LRRC8C-8A (IL1^25^)) that lacks an arginine at the equivalent position of LRRC8A(R103) is strongly inhibited by DCPIB. These observations raise concerns about the utility of LRRC8A homomeric channel structures in guiding mutagenesis-based analysis of structure-function-pharmacology relationships.

The use of LRRC8C-8A (IL1^25^) or other chimeras provides several important advantages over LRRC8A for structure–function studies of VRAC regulation and pharmacology. First, LRRC8C-8A (IL1^25^) channels exhibit native-like activation by low intracellular ionic strength and cell swelling, and inactivation by hypertonic cell shrinkage. Second, their DCPIB sensitivity closely resembles that of native VRAC currents. Third, and importantly, they form homomeric channels that circumvent complicating issues with subunit stoichiometry and position described above. Solving the structure of LRRC8C-8A (IL1^25^) could provide a more “physiological” model for understanding how VRAC channels are regulated by cell volume changes and modulated pharmacologically.

## Conclusions

The discovery of the *LRRC8* gene family that encodes VRAC has generated renewed interest in understanding the molecular physiology and therapeutic potential of these channels in human diseases. Despite proving useful in the initial characterizations of VRAC physiology, its pharmacology remains underdeveloped. DCPIB was initially described as a VRAC inhibitor 20 years ago and is still the best-in-class inhibitor to date, even with its myriad of off-targets. Our lab has only begun to better understand how native VRAC may be modulated by small-molecule compounds utilizing the robust YFP-quenching assays previously established as a functional readout of chloride channel activity [72,82]. Others have used cryo-EM to identify putative binding sites of small-molecule VRAC inhibitors and using the gained structural insight to develop even more potent channel blockers [12,22]. Protein engineering approaches have generated LRRC8 chimeras that may serve as a complementary approach to identify putative binding sites of known VRAC modulators [83]. Furthermore, these chimeras could be used to study the pharmacological modulation of individual LRRC8 subunits. Given the emerging genetic knockout evidence that certain VRAC subunits subtypes (i.e. LRRC8C-E) play specific physiological roles, subunit-specific modulators would be useful in further testing these hypotheses. While VRAC’s pharmacology is still in its infancy, ongoing studies are paving the way to generate tools to study these important channels and ultimately test if targeting VRAC is therapeutic for human disease.

## References

[1] Churchwell KB, Wright SH, Emma F, et al. NMDA receptor activation inhibits neuronal volume regulation after swelling induced by veratridine-stimulated Na+ influx in rat cortical cultures. J Neurosci. 1996;16(23):7447–7457. 8922400 10.1523/JNEUROSCI.16-23-07447.1996 PMC6579111

[2] Strange K. Cellular volume homeostasis. Adv Physiol Educ. 2004;28(1–4):155–159. 15545344 10.1152/advan.00034.2004

[3] Hoffmann EK, Lambert IH, Pedersen SF. Physiology of cell volume regulation in vertebrates. Physiol Rev. 2009;89(1):193–277. 19126758 10.1152/physrev.00037.2007

[4] Lang F, Lepple-Wienhues A, Paulmichl M, et al. Ion channels, cell volume, and apoptotic cell death. Cell Physiol Biochem. 1998;8:285–292. 9949254 10.1159/000016290

[5] Wehner F, Olsen H, Tinel H, et al. Cell volume regulation: osmolytes, osmolyte transport, and signal transduction. Rev Physiol Biochem Pharmacol. 2003;148:1–80. 12687402 10.1007/s10254-003-0009-x

[6] Evans DH. Osmotic and ionic regulation: cells and animals. Boca Raton: CRC Press; 2009. p. xvi, 590, 8 of plates.

[7] Strange K, Yamada T, Denton JS. A 30-year journey from volume-regulated anion currents to molecular structure of the LRRC8 channel. J Gen Physiol. 2019;151(2):100–117. 30651298 10.1085/jgp.201812138 PMC6363415

[8] Voss FK, Ullrich F, Münch J, et al. Identification of LRRC8 heteromers as an essential component of the volume-regulated anion channel VRAC. Science. 2014;344(6184):634–638. 24790029 10.1126/science.1252826

[9] Qiu Z, Dubin A, Mathur J, et al. SWELL1, a plasma membrane protein, is an essential component of volume-regulated anion channel. Cell. 2014;157(2):447–458. 24725410 10.1016/j.cell.2014.03.024 PMC4023864

[10] Abascal F, Zardoya R. LRRC8 proteins share a common ancestor with pannexins, and may form hexameric channels involved in cell-cell communication. Bioessays. 2012;34(7):551–560. 22532330 10.1002/bies.201100173

[11] Nakamura R, Numata T, Kasuya G, et al. Cryo-EM structure of the volume-regulated anion channel LRRC8D isoform identifies features important for substrate permeation. Commun Biol. 2020;3(1):240. 32415200 10.1038/s42003-020-0951-z PMC7229184

[12] Kern DM, Oh S, Hite RK, Brohawn SG . Cryo-EM structures of the DCPIB-inhibited volume-regulated anion channel LRRC8A in lipid nanodiscs. Elife. 2019;8:e42636. doi: 10.7554/eLife.42636. PMC6395065 30775971

[13] Kasuya G, Nakane T, Yokoyama T, et al. Cryo-EM structures of the human volume-regulated anion channel LRRC8. Nat Struct Mol Biol. 2018;25(9):797–804. 30127360 10.1038/s41594-018-0109-6

[14] Kefauver JM, Saotome K, Dubin AE, et al. Structure of the human volume regulated anion channel. Elife. 2018;7. DOI:10.7554/eLife.38461 PMC6086657 30095067

[15] Deneka D, Sawicka M, Lam AKM, et al. Structure of a volume-regulated anion channel of the LRRC8 family. Nature. 2018;558(7709):254–259. 29769723 10.1038/s41586-018-0134-y

[16] Zhou C, Chen X, Planells-Cases R, et al. Transfer of cGAMP into bystander cells via LRRC8 volume-regulated anion channels augments STING-mediated interferon responses and anti-viral immunity. Immunity. 2020;52(5):767–781 e6. 32277911 10.1016/j.immuni.2020.03.016

[17] Lahey LJ, Mardjuki RE, et al. The LRRC8A:C heteromeric channel is a cGAMP Transporter and the Dominant cGAMP importer in human vasculature cells. bioRxiv. 2020;2020.02.13.948273.

[18] Alghanem AF, Abello J, et al. The SWELL1-LRRC8 complex regulates endothelial AKT-eNOS-mTOR signaling and vascular function. bioRxiv. 2020;2020.08.04.236182. 10.7554/eLife.61313 PMC7997661 33629656

[19] Osei-Owusu J, Yang J, et al. Molecular biology and physiology of volume-regulated anion channel (VRAC). Curr Top Membr. 2018;81:177–203. 30243432 10.1016/bs.ctm.2018.07.005 PMC6604840

[20] Chen L, König B, Liu T, et al. More than just a pressure relief valve: physiological roles of volume-regulated LRRC8 anion channels. Biol Chem. 2019;400(11):1481–1496. 31091194 10.1515/hsz-2019-0189

[21] Gunasekar SK, Xie L, Sah R. SWELL signalling in adipocytes: can fat ‘feel’ fat? Adipocyte. 2019;8(1):223–228. 31112068 10.1080/21623945.2019.1612223 PMC6768237

[22] Gunasekar SK, Litao Xie, et al. Small molecule SWELL1-LRRC8 complex induction improves glycemic control and nonalcoholic fatty liver disease in murine Type 2 diabetes. bioRxiv. 2021;2021.02.28.432901. 10.1038/s41467-022-28435-0 PMC8831520 35145074

[23] Knauf PA, Rothstein A. Chemical modification of membranes. II. Permeation paths for sulfhydryl agents. J Gen Physiol. 1971;58(2):211–223. 5559623 10.1085/jgp.58.2.211 PMC2226013

[24] Russell JM, Boron WF. Role of choloride transport in regulation of intracellular pH. Nature. 1976;264(5581):73–74. 12472 10.1038/264073a0

[25] Russell JM, Brodwick MS. Properties of chloride transport in barnacle muscle fibers. J Gen Physiol. 1979;73(3):343–368. 438775 10.1085/jgp.73.3.343 PMC2215166

[26] White MM, Miller C. A voltage-gated anion channel from the electric organ of Torpedo californica. J Biol Chem. 1979;254(20):10161–10166. 489590

[27] Ehrenspeck G, Brodsky WA. Effects of 4-acetamido-4’-isothiocyano-2,2-disulfonic stilbene on ion transport in turtle bladders. Biochim Biophys Acta. 1976;419(3):555–558. 129157 10.1016/0005-2736(76)90268-6

[28] Kubo M, Okada Y. Volume-regulatory Cl- channel currents in cultured human epithelial cells. J Physiol. 1992;456:351–371. 1284079 10.1113/jphysiol.1992.sp019340 PMC1175685

[29] Dick GM, Kong ID, Sanders KM. Effects of anion channel antagonists in canine colonic myocytes: comparative pharmacology of Cl-, Ca2+ and K+ currents. Br J Pharmacol. 1999;127(8):1819–1831. 10482912 10.1038/sj.bjp.0702730 PMC1566175

[30] Helix N, Strabaek D, Dahl BH, et al. Inhibition of the endogenous volume-regulated anion channel (VRAC) in HEK293 cells by acidic di-aryl-ureas. J Membr Biol. 2003;196(2):83–94. 14724745 10.1007/s00232-003-0627-x

[31] Stott JB, deCourcey F, Ennis M, et al. Functional and pharmacological characterization of volume-regulated anion channels in human normal and cystic fibrosis bronchial and nasal epithelial cells. Eur J Pharmacol. 2014;740:183–191. 25034811 10.1016/j.ejphar.2014.07.007

[32] Boese SH, Kinne RHK, Wehner F. Single-channel properties of swelling-activated anion conductance in rat inner medullary collecting duct cells. Am J Physiol (Renal Fluid Electrolyte Physiol.40). 1996;271:F1224–F1233. 10.1152/ajprenal.1996.271.6.F1224 8997397

[33] Sato-Numata K, Numata T, Inoue R, et al. Distinct pharmacological and molecular properties of the acid-sensitive outwardly rectifying (ASOR) anion channel from those of the volume-sensitive outwardly rectifying (VSOR) anion channel. Pflugers Arch. 2016;468(5):795–803. 26743872 10.1007/s00424-015-1786-1

[34] Bakhramov A, Fenech C, Bolton TB. Chloride current activated by hypotonicity in cultured human astrocytoma cells. Exp Physiol. 1995;80(3):373–389. 7543762 10.1113/expphysiol.1995.sp003854

[35] Decher N, Lang HJ, Nilius B, et al. DCPIB is a novel selective blocker of ICl,swell and prevents swelling-induced shortening of Guinea-pig atrial action potential duration. Br J Pharmacol. 2001;134(7):1467–1479. 11724753 10.1038/sj.bjp.0704413 PMC1573095

[36] Abdullaev IF, Rudkouskaya A, Schools GP, et al. Pharmacological comparison of swelling-activated excitatory amino acid release and Cl− currents in cultured rat astrocytes. J Physiol. 2006;572(Pt 3):677–689. 16527858 10.1113/jphysiol.2005.103820 PMC1780004

[37] Akita T, Okada Y. Regulation of bradykinin-induced activation of volume-sensitive outwardly rectifying anion channels by Ca2+ nanodomains in mouse astrocytes. J Physiol. 2011;589(Pt 16):3909–3927. 21690189 10.1113/jphysiol.2011.208173 PMC3179992

[38] Harrigan TJ, Abdullaev IF, et al. Activation of microglia with zymosan promotes excitatory amino acid release via volume-regulated anion channels: the role of NADPH oxidases. J Neurochem. 2008;106(6):2449–2462. 18624925 10.1111/j.1471-4159.2008.05553.x PMC2574595

[39] Bantel C, Maze M, Trapp S. Neuronal preconditioning by inhalational anesthetics: evidence for the role of plasmalemmal adenosine triphosphate-sensitive potassium channels. Anesthesiology. 2009;110(5):986–995. 19352153 10.1097/ALN.0b013e31819dadc7 PMC2930813

[40] Schlichter LC, Mertens T, Liu B. Swelling activated Cl- channels in microglia: biophysics, pharmacology and role in glutamate release. Channels (Austin). 2011;5(2):128–137. 21150294 10.4161/chan.5.2.14310 PMC3127054

[41] Yamada T, Figueroa EE, et al. LRRC8A homohexameric channels poorly recapitulate VRAC regulation and pharmacology. Am J Physiol Cell Physiol. 2021;320(3):C293–C303. 33356947 10.1152/ajpcell.00454.2020 PMC8294627

[42] Sabirov RZ, Okada Y. ATP release via anion channels. Purinergic Signal. 2005;1(4):311–328. 18404516 10.1007/s11302-005-1557-0 PMC2096548

[43] Sabirov RZ, Merzlyak PG, et al. The properties, functions, and pathophysiology of maxi-anion channels. Pflugers Arch. 2016;468(3):405–420. 26733413 10.1007/s00424-015-1774-5

[44] Afzal A, Figueroa EE, Kharade SV, et al. The LRRC8 volume-regulated anion channel inhibitor, DCPIB, inhibits mitochondrial respiration independently of the channel. Physiol Rep. 2019;7(23):e14303. 31814333 10.14814/phy2.14303 PMC6900491

[45] Bowens NH, Dohare P, Kuo Y-H, et al. DCPIB, the proposed selective blocker of volume-regulated anion channels, inhibits several glutamate transport pathways in glial cells. Mol Pharmacol. 2013;83(1):22–32. 23012257 10.1124/mol.112.080457 PMC3533478

[46] Minieri L, Pivonkova H, Caprini M, et al. The inhibitor of volume-regulated anion channels DCPIB activates TREK potassium channels in cultured astrocytes. Br J Pharmacol. 2013;168(5):1240–1254. 23072356 10.1111/bph.12011 PMC3594680

[47] Fujii T, Takahashi Y, Takeshima H, et al. Inhibition of gastric H+,K+-ATPase by 4-(2-butyl-6,7-dichloro-2-cyclopentylindan-1-on-5-yl)oxybutyric acid (DCPIB), an inhibitor of volume-regulated anion channel. Eur J Pharmacol. 2015;765:34–41. 26277321 10.1016/j.ejphar.2015.08.011

[48] Deng W, Mahajan R, Baumgarten CM, et al. The ICl,swell inhibitor DCPIB blocks Kir channels that possess weak affinity for PIP2. Pflugers Arch. 2016;468(5):817–824. 26837888 10.1007/s00424-016-1794-9 PMC5317042

[49] Kurbannazarova RS, Bessonova SV, Okada Y, et al. Swelling-activated anion channels are essential for volume regulation of mouse thymocytes. Int J Mol Sci. 2011;12(12):9125–9137. 22272123 10.3390/ijms12129125 PMC3257120

[50] Klausen TK, Bergdahl A, Hougaard C, et al. Cell cycle-dependent activity of the volume- and Ca2+-activated anion currents in Ehrlich lettre ascites cells. J Cell Physiol. 2007;210(3):831–842. 17111356 10.1002/jcp.20918

[51] Fan HT, Morishima S, Kida H, et al. Phloretin differentially inhibits volume-sensitive and cyclic AMP-activated, but not Ca-activated, Cl − channels. Br J Pharmacol. 2001;133(7):1096–1106. 11487521 10.1038/sj.bjp.0704159 PMC1572865

[52] Zhang J, Dawson VL, Dawson TM, et al. Nitric oxide activation of poly(ADP-ribose) synthetase in neurotoxicity. Science. 1994;263:687–689. 8080500 10.1126/science.8080500

[53] Tominaga M, Tominaga T, Miwa A, et al. Volume-sensitive chloride channel activity does not depend on endogenous P-glycoprotein. J Biol Chem. 1995;270(46):27887–27893. 7499263 10.1074/jbc.270.46.27887

[54] Hechenberger M, Schwappach B, Fischer WN, et al. A family of putative chloride channels from Arabidopsis and functional complementation of a yeast strain with a CLC gene disruption. J Biol Chem. 1996;271(52):33632–33638. 8969232 10.1074/jbc.271.52.33632

[55] Wondergem R, Gong W, Monen SH, et al. Blocking swelling-activated chloride current inhibits mouse liver cell proliferation. J Physiol. 2001;532:661–672. 11313437 10.1111/j.1469-7793.2001.0661e.x PMC2278564

[56] Chen L, Wang L, et al. Cell cycle-dependent expression of volume-activated chloride currents in nasopharyngeal carcinoma cells. Am J Physiol Cell Physiol. 2002;283(4):C1313–23. 12225994 10.1152/ajpcell.00182.2002

[57] Yang X, Zhu L, et al. Cisplatin activates volume-sensitive like chloride channels via purinergic receptor pathways in nasopharyngeal carcinoma cells. J Membr Biol. 2015;248(1):19–29. 25236172 10.1007/s00232-014-9724-2

[58] Leaney JL, Marsh SJ, Brown DA. A swelling-activated chloride current in rat sympathetic neurones. J Physiol. 1997;501(3):555–564. 9218216 10.1111/j.1469-7793.1997.555bm.x PMC1159457

[59] Zhang H, Cao HJ, et al. Volume regulated anion channel currents of rat hippocampal neurons and their contribution to oxygen-and-glucose deprivation induced neuronal death. PLoS One. 2011;6(2):e16803. 21347298 10.1371/journal.pone.0016803 PMC3037944

[60] Inoue H, Mori S-I, Morishima S, et al. Volume-sensitive chloride channels in mouse cortical neurons: characterization and role in volume regulation. Eur J Neurosci. 2005;21(6):1648–1658. 15845092 10.1111/j.1460-9568.2005.04006.x

[61] Benfenati V, Caprini M, Nicchia GP, et al. Carbenoxolone inhibits volume-regulated anion conductance in cultured rat cortical astroglia. Channels (Austin). 2009;3(5):323–336. 19713739 10.4161/chan.3.5.9568

[62] Gaitan-Penas H, Gradogna A, et al. Investigation of LRRC8-mediated volume-regulated anion currents in xenopus oocytes. Biophys J. 2016;111(7):1429–1443. 27705766 10.1016/j.bpj.2016.08.030 PMC5052465

[63] Jayaraman S, Haggie P, Wachter RM, et al. Mechanism and cellular applications of a green fluorescent protein-based halide sensor. J Biol Chem. 2000;275(9):6047–6050. 10692389 10.1074/jbc.275.9.6047

[64] Galietta LJ, Haggie PM, Verkman AS. Green fluorescent protein-based halide indicators with improved chloride and iodide affinities. FEBS Lett. 2001;499(3):220–224. 11423120 10.1016/s0014-5793(01)02561-3

[65] Tertyshnikova S, Dworetzky S, et al. Cell-based assay for the quantitative high throughput screening of gamma-aminobutyric acid-induced halide transport. Google Patents. 2006.

[66] Verkman AS, Galietta LJ. Chloride channels as drug targets. Nat Rev Drug Discov. 2009;8(2):153–171. 19153558 10.1038/nrd2780 PMC3601949

[67] Yamaguchi T, Kohrogi H, Honda I, et al. A novel leukotriene antagonist, ONO-1078, Inhibits and reverses human bronchial contraction induced by leukotrienes C4 and D 4 and antigen in vitro. Am Rev Respir Dis. 1992;146(4):923–929. 1416420 10.1164/ajrccm/146.4.923

[68] Yamaguchi T, Kohrogi H, Honda I, et al. Preventive effect of a novel leukotrienes antagonist ONO-1078 on leukotriene C4- and D4-induced human bronchial smooth muscle contraction. Arerugi. 1990;39(11):1477–1483. 2288493

[69] Heise CE, O’Dowd BF, Figueroa DJ, et al. Characterization of the human cysteinyl leukotriene 2 receptor. J Biol Chem. 2000;275(39):30531–30536. 10851239 10.1074/jbc.M003490200

[70] Lynch KR, O’Neill GP, Liu Q, et al. Characterization of the human cysteinyl leukotriene CysLT1 receptor. Nature. 1999;399(6738):789–793. 10391245 10.1038/21658

[71] Holm JB, Grygorczyk R, Lambert IH. Volume-sensitive release of organic osmolytes in the human lung epithelial cell line A549: role of the 5-lipoxygenase. Am J Physiol Cell Physiol. 2013;305(1):C48–60. 23485709 10.1152/ajpcell.00412.2012

[72] Figueroa EE, Kramer M, Strange K, et al. CysLT1 receptor antagonists pranlukast and zafirlukast inhibit LRRC8-mediated volume regulated anion channels independently of the receptor. Am J Physiol Cell Physiol. 2019;317(4):C857–C866. 31390227 10.1152/ajpcell.00281.2019 PMC6850990

[73] Mo J, Lin D, Wang J, et al. Apoptosis in HepG2 cells induced by zinc pyrithione via mitochondrial dysfunction pathway: involvement of zinc accumulation and oxidative stress. Ecotoxicol Environ Saf. 2018;161:515–525. 29913420 10.1016/j.ecoenv.2018.06.026

[74] Carraway RE, Dobner PR. Zinc pyrithione induces ERK- and PKC-dependent necrosis distinct from TPEN-induced apoptosis in prostate cancer cells. Biochim Biophys Acta. 2012;1823(2):544–557. 22027089 10.1016/j.bbamcr.2011.09.013

[75] Shimizu T, Numata T, Okada Y. A role of reactive oxygen species in apoptotic activation of volume-sensitive Cl(-) channel. Proc Natl Acad Sci U S A. 2004;101(17):6770–6773. 15096609 10.1073/pnas.0401604101 PMC404120

[76] Jiao J-D, Xu C-Q, Yue P, et al. Volume-sensitive outwardly rectifying chloride channels are involved in oxidative stress-induced apoptosis of mesangial cells. Biochem Biophys Res Commun. 2006;340(1):277–285. 16364252 10.1016/j.bbrc.2005.11.175

[77] Browe DM, Baumgarten CM. Angiotensin II (AT1) receptors and NADPH oxidase regulate Cl- current elicited by beta1 integrin stretch in rabbit ventricular myocytes. J Gen Physiol. 2004;124(3):273–287. 15337822 10.1085/jgp.200409040 PMC2233887

[78] Gradogna A, Gavazzo P, Boccaccio A, et al. Subunit-dependent oxidative stress sensitivity of LRRC8 volume-regulated anion channels. J Physiol. 2017;595(21):6719–6733. 28841766 10.1113/JP274795 PMC5663833

[79] Tailler M, Senovilla L, Lainey E, et al. Antineoplastic activity of ouabain and pyrithione zinc in acute myeloid leukemia. Oncogene. 2012;31(30):3536–3546. 22105358 10.1038/onc.2011.521

[80] Srivastava G, Matta A, et al. Anticancer activity of pyrithione zinc in oral cancer cells identified in small molecule screens and xenograft model: implications for oral cancer therapy. Mol Oncol. 2015;9(8):1720–1735. 26115765 10.1016/j.molonc.2015.05.005 PMC5528795

[81] Okada Y, Numata T, Sato-Numata K, et al. Roles of volume-regulatory anion channels, VSOR and Maxi-Cl, in apoptosis, cisplatin resistance, necrosis, ischemic cell death, stroke and myocardial infarction. Curr Top Membr. 2019;83:205–283. 31196606 10.1016/bs.ctm.2019.03.001

[82] Figueroa EE, Denton JS. Zinc pyrithione activates the volume-regulated anion channel through an antioxidant-sensitive mechanism. Am J Physiol Cell Physiol. 2021;320(6):C1088–C1098. 33826406 10.1152/ajpcell.00070.2021 PMC8285639

[83] Yamada T, Strange K. Intracellular and extracellular loops of LRRC8 are essential for volume-regulated anion channel function. J Gen Physiol. 2018;150(7):1003–1015. 29853476 10.1085/jgp.201812016 PMC6028502

